# Exogenous Nitric Oxide Mitigates Nickel-Induced Oxidative Damage in Eggplant by Upregulating Antioxidants, Osmolyte Metabolism, and Glyoxalase Systems

**DOI:** 10.3390/plants8120562

**Published:** 2019-12-01

**Authors:** Mona Soliman, Haifa A. Alhaithloul, Khalid Rehman Hakeem, Basmah M. Alharbi, Mohamed El-Esawi, Amr Elkelish

**Affiliations:** 1 Botany and Microbiology Department, Faculty of Science, Cairo University, Giza 12613, Egypt; monahsh1@gmail.com; 2 Department of Biology, College of science, Jouf University, Sakaka 2014, Saudi Arabia; haifasakat2030@gmail.com; 3 Department of Biological Sciences, Faculty of Science, King Abdulaziz University, Jeddah 21577, Saudi Arabia; 4 Princess Dr Najla Bint Saud Al- Saud Center for Excellence Research in Biotechnology, King Abdulaziz University, P.O. Box 80200, Jeddah, Saudi Arabia; 5 Department of Biology, Faculty of Science, University of Tabuk, Tabuk 71491, Saudi Arabia; b.alharbi@ut.edu.sa; 6 Botany Department, Faculty of Science, Tanta University, Tanta 31527, Egypt; mohamed.elesawi@science.tanta.edu.eg; 7 Sainsbury Laboratory, University of Cambridge, Cambridge CB2 1LR, UK; 8 Botany Department, Faculty of Science, Suez Canal University, Ismailia 41522, Egypt; amr.elkelish@science.suez.edu.eg

**Keywords:** Antioxidants, Glyoxalase, Lipid peroxidation, Lipoxygenase, Nickel, Nitric oxide

## Abstract

Nitric oxide (NO) at optimal levels is considered beneficial to plant functioning. The present study was carried out to investigate the role of exogenously applied NO (100 and 150 µM sodium nitropurusside, SNP) in amelioration of nickel (Ni)-mediated oxidative effects in eggplant. Ni stress declined growth and biomass production, relative water content (RWC), and chlorophyll pigment synthesis, thereby affecting the photosynthetic efficiency. Exogenously applied SNP proved beneficial in mitigating the Ni-mediated growth restrictions. NO-treated seedlings exhibited improved photosynthesis, stomatal conductance, and chlorophyll content with the effect of being apparent at lower concentration (100 µM SNP). SNP upregulated the antioxidant system mitigating the oxidative damage on membranes due to Ni stress. The activity of superoxide dismutase, catalase, glutathione S-transferase, ascorbate peroxidase, and glutathione reductase was upregulated due to SNP which also increased the ascorbate and reduced glutathione content. SNP-supplied seedlings also showed higher proline and glycine betaine accumulation, thereby improving RWC and antioxidant system. Glyoxalase I activity was induced due to SNP application declining the accumulation of methylglyoxal. NO-mediated mitigation of Ni toxicity was confirmed using NO scavenger (PTIO, 2-phenyl-4,4,5,5-tetramethylimidazoline-1-oxyl-3-oxide), which reversed the influence of SNP almost entirely on the parameters studied. Uptake of nitrogen (N), potassium (K), and calcium (Ca) was increased due to SNP application and Ni was reduced significantly. Therefore, this study revealed the efficiency of exogenous SNP in enhancing Ni stress tolerance through upregulating antioxidant and glyoxalase systems.

## 1. Introduction

Accumulation of heavy metals in agricultural land significantly reduces soil fertility and the yield potential of crop plants, thereby imparting threat to global food security. Once taken up by plants, these toxic metals accumulate in the food chain, thereby influencing the health of animals, including humans [1]. Among the key toxic metals, the pollutant nickel is added to the soil from various anthropogenic sources like disposal of Ni-Cd batteries, mining, smelting, and electroplating industry [1,2,3]. Relatively low levels, i.e., when accumulated in the range of 0.01 to 5 μg g^−1^ dry weight Ni is considered to promote plant growth and development [4]; however, at higher concentrations, it severely affects the growth and development by imparting deleterious effects on photosynthesis, enzyme activity, and mineral nutrition [5]. Metal toxicity imbalances cellular redox homeostasis and mineral uptake, inhibits photosynthesis, damage nucleic acids, and mediate protein oxidation [6]. These damaging effects result from the excessive reactive oxygen species (ROS) thereby imparting oxidative damage, and toxic metals have been reported to alter the phytohormone profile, chloroplast structure and functioning [7,8]. To mitigate these adverse effects of metal toxicity, plants upregulate the indigenous tolerance mechanisms including antioxidant and osmolyte metabolism, and glyoxylase system [9]. The antioxidant system is comprised of enzymatic and nonenzymatic components, which neutralize excess ROS [10], and osmolytes, including proline, glycine betaine (GB), sugars, etc., leading to protection of water relations and enzyme activity [11]. Another important mechanism i.e., glyoxalase system includes two key enzymes: glyoxalase I and II mediating the detoxification of methylglyoxal (MG) [9]. Exogenous supplementation of phytohormones strengthen the tolerance mechanisms and improve tolerance of crop plants to a variety of stresses [5,8,12,13,14].

Nitric oxide (NO) is considered to actively regulate several vital physiological functions including germination, growth, enzyme activity, responses to stresses and stress signaling [15,16]. Exogenous application of NO prevents the salinity-induced photosynthetic inhibition by neutralizing the accumulated ROS [17,18,19]. However, the excess can be toxic due to its radical nature. Therefore, supplementation of the optimal concentration of NO can substantially alleviate the damaging effects of stresses by triggering tolerance mechanisms. In salt-stressed *Brassica juncea*, it has been reported that NO mitigates the oxidative effects on growth by improving antioxidant functioning and osmolyte accumulation resulting in enhanced chlorophyll synthesis and carbonic anhydrase activity [20,21].

Recent studies have shown that due to lack of proper disposal and damping facility for industrial wastes the concentrations of several toxic heavy metals is increasing in soils of Egypt [22]. Eggplant is an important vegetable crop plant grown worldwide. It is rich in antioxidants like anthocyanins, which include nasunin, lutein, and zeaxanthin, and chlorogenic acid, vitamins, and minerals. Increased Ni availability in the soil can affect eggplant productivity considerably and can have deleterious effects on human health as well. Therefore, we envisaged that exogenous application of NO can reduce the Ni stress-mediated damage to growth and development of eggplant by influencing the key tolerance mechanisms including antioxidants, osmolytes, and glyoxylase system. To test this hypothesis, pot experiments were conducted as described below.

## 2. Results

### 2.1. Exogenous SNP Improves Growth by Enhancing Mineral Uptake and Reducing Ni Accumulation

Results showing the effect of Ni stress on height and dry weight of eggplant with and without exogenous application of SNP are shown in Table 1. Relative to control, applied SNP increased plant height and dry weight and ameliorated the reduction induced by Ni treatment. Maximal enhancement of 15.81% in height and 29.80% in dry weight was observed in 100 µM NO-treated plants and amelioration of 17.86% and 46.15% was observed in plants treated with Ni + 100 µM SNP over the Ni-stressed plants. However, 150 µM SNP did not prove much beneficial compared to control, but maintained relatively increased growth over Ni-stressed counterparts (Table 1). Moreover, the content of N, K, and Ca also registered an apparent enhancement, due to SNP application with maximal effect with 100 µM concentration. Relative to control, percent decline in N, K and Ca was 44.23, 44.25, and 48.55%, respectively, in Ni-stressed plants; amelioration of 29.07, 26.80, and 32.66% with Ni + 100 µM SNP and 14.56, 16.43, and 11.66% with Ni + 150 µM SNP was observed (Table 1). Exogenous application of SNP significantly declined the accumulation of Ni in leaf tissues. Percent decline in Ni uptake was maximum (30.42%) in plants treated with Ni + 100 µM SNP (Table 1).

### 2.2. Photosynthetic Pigments, Photosynthesis, and Gas Exchange Parameters Increased Due to Exogenous SNP

Treatment of Ni reduced the chlorophyll and carotenoid, significantly resulting in declined net photosynthesis, stomatal conductance, and intercellular CO_2_. Ni declined total chlorophylls by 40.69%, carotenoids by 35.40%, net photosynthesis by 46.01%, stomatal conductance by 25.35%, and intercellular CO_2_ by 26.29% over the control plants. Application of SNP at both concentrations increased the pigment and gas exchange parameters with maximal increase and amelioration at 100 µM SNP. Increase in total chlorophylls, carotenoids, net photosynthesis, stomatal conductance, and intercellular CO_2_ was 28.40, 18.60, 34.80, 25.03, and 30.75%, respectively, due to 100 µM SNP, and this effect was maintained when supplied to Ni-stressed plants exhibiting an amelioration of 29.82% for total chlorophyll, 30.86% for carotenoids, 33.83% for net photosynthesis, 25.21% for stomatal conductance, and 223.66% for intercellular CO_2_ in Ni + 100 µM SNP (Table 2).

### 2.3. NO Supplemented Seedlings Exhibit Higher RWC, Proline, and Glycine Betaine (GB)

**Figure 1:**
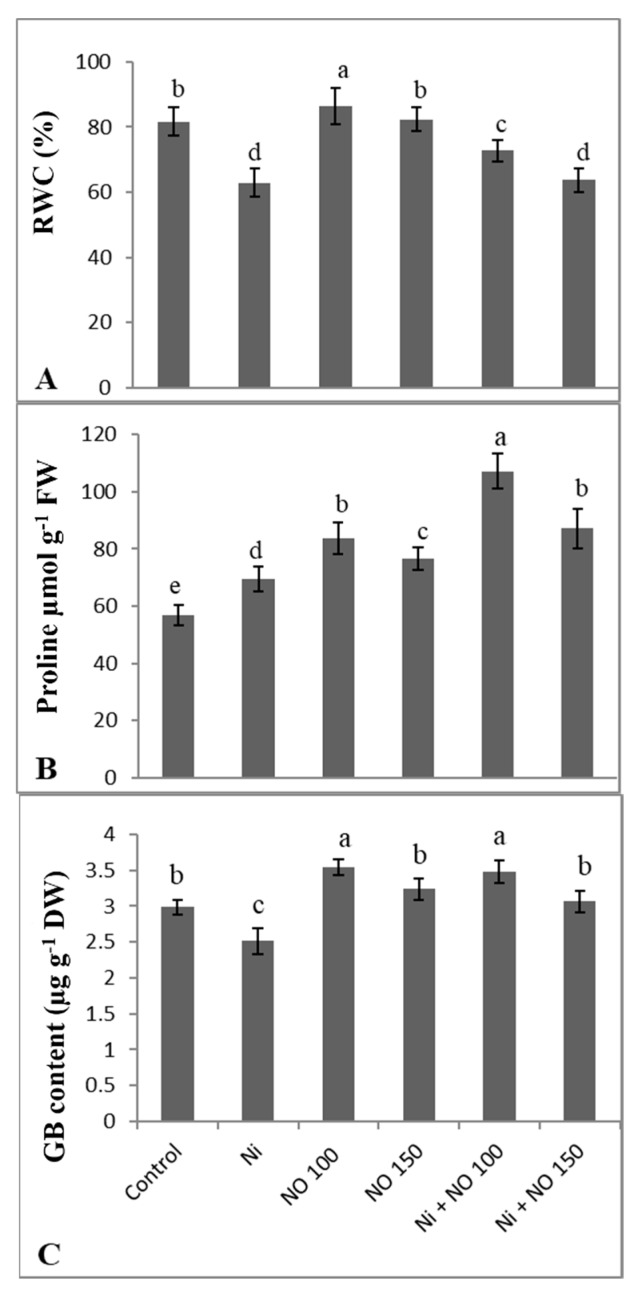
Effect of Ni (100 mg kg^−1^ soil) on (A) relative water content, (B) proline and (C) glycine betaine in eggplant (*Solanum melongena* L.) treated with nitric oxide (100 and 150 µM SNP). Data presented is mean (±SE) of three replicates and bars with different letters denote significant difference at *p* ≤ 0.05. Control = 0 mg kg^−1^ soil.

Seedlings grown on Ni supplemented soil exhibited a decline of 22.88% in RWC; however, an increase of 5.49% and 0.48%, due to 100 and 150 µM SNP application, was observed over the control (Figure 1A). Application of 100 µM SNP significantly ameliorate the decline in RWC. Under normal conditions, the exogenous application of 100 µM SNP increased the content of proline and GB by 31.85 and 15.81% over the control. Per cent increase in proline (18.24%) in Ni-stressed plants was further enhanced by 35.01% due to 100 µM SNP application. GB decreased (15.77%) because Ni stress and the application of 100 µM SNP enhanced GB content by 27.87% over the Ni-stressed plants (Figure 1B,C).

### 2.4. Application of SNP Reduces Oxidative Damage by Declining H_2_O_2_, Lipid Peroxidation, and Lipoxygenase

**Figure 2:**
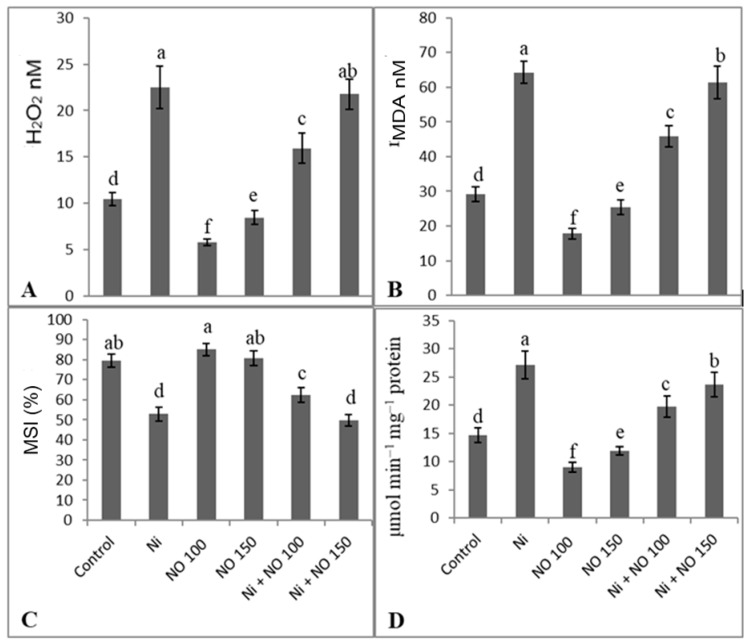
Effect of Ni (100 mg kg^−1^ soil) on (**A**) hydrogen peroxide (**B**) lipid peroxidation (MDA), (**C**) membrane stability index (MSI) and (**D**) lipoxygenase activity in eggplant (*Solanum melongena* L.) treated with nitric oxide (100 and 150 µM SNP). Data presented is mean (±SE) of three replicates and bars with different letters denote significant difference at *p* ≤ 0.05. Control = 0 mg kg^−1^ soil.

Ni stress caused oxidative damage by triggering the accumulation of H_2_O_2_ (53.53%), resulting in increased lipid peroxidation (54.65%) and declined MSI (33.31). However, the exogenous application of SNP (100 µM) significantly declined the generation of H_2_O_2_ by 44.92%, causing a decrease of 38.95% in lipid peroxidation and increase of MSI by 6.80% (Figure 2A–C). A significant reduction was observed in H_2_O_2_ generation and lipid peroxidation when SNP (100 µM) was applied to Ni-stressed plants. Also, SNP-treated seedlings showed an apparent decline in lipoxygenase activity with 38.31 and 18.98% decline at 100 and 150 µM NO (Figure 2D). Relative to control, lipoxygenase activity increased by 45.89%, due to Ni treatment, and was maximally ameliorated by 27.08% due to 100 µM SNP application (Figure 2D). 

### 2.5. NO Upregulates Antioxidant System under Ni Stress

**Figure 3:**
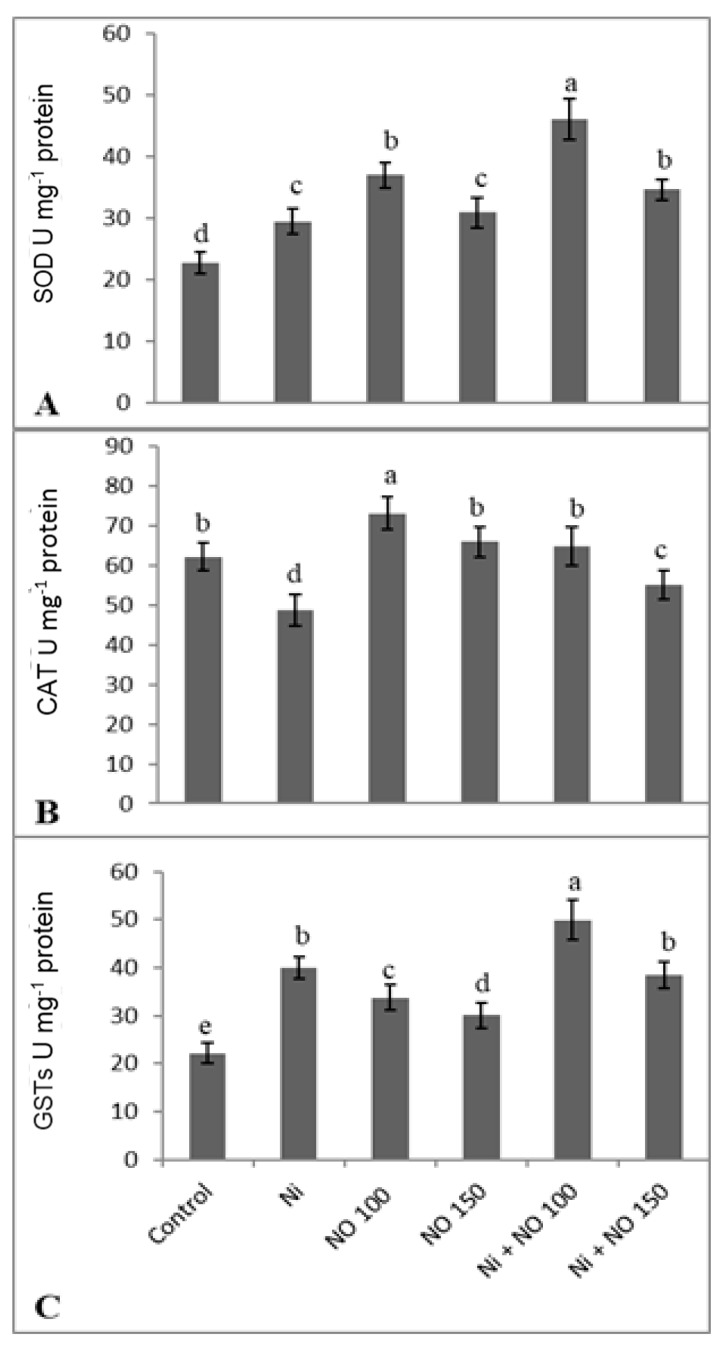
Effect of Ni (100 mg kg^−1^ soil) on the activity of (**A**) superoxide dismutase (SOD), (**B**) catalase (CAT) and (**C**) glutathione S-transferase (GSTs) in eggplant (*Solanum melongena* L.) treated with nitric oxide (100 and 150 µM SNP). Data presented is mean (±SE) of three replicates and bars with different letters denote significant difference at *p* ≤ 0.05. Control = 0 mg kg^−1^ soil.

**Figure 4:**
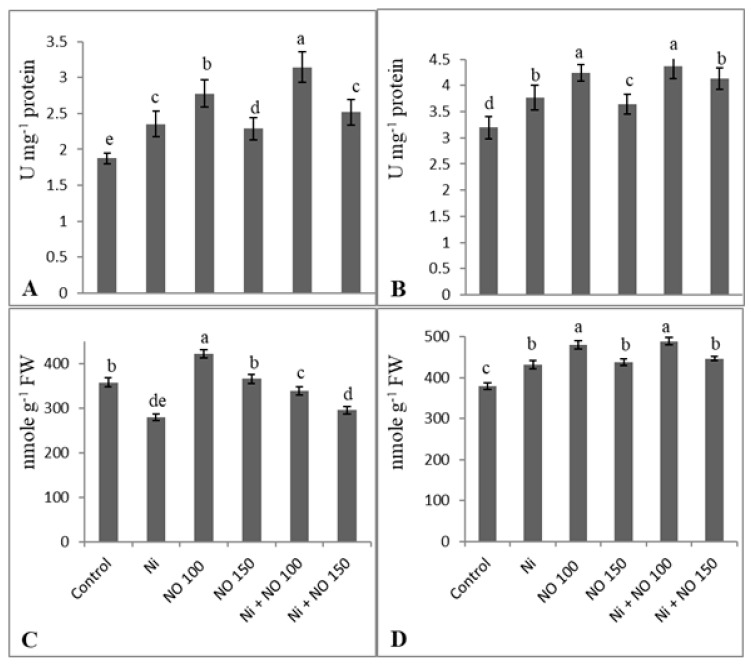
Effect of Ni (100 mg kg^−1^ soil) on the activity of (**A**) ascorbate peroxidase and (**B**) glutathione reductase and content of (**C**) ascorbate and (**D**) reduced glutathione in eggplant (*Solanum melongena* L.) treated with nitric oxide (100 and 150 µM SNP). Data presented is mean (±SE) of three replicates and bars with different letters denote significant difference at *p* ≤ 0.05. Control = 0 mg kg^−1^ soil.

SNP application upregulated the antioxidant system and enhancement was much apparent in seedlings supplied with 100 µM SNP than 150 µM SNP and Ni-stressed ones. Relative to control, activities of SOD, CAT, GST, APX, and GR increased maximally by 50.78, 4.09, 55.68, 40.44, and 27.00%, respectively in Ni + 100 µM SNP-treated seedlings. Relative to control activities of SOD (23.00%), GST (44.60%), APX (20.42%), and GR (15.15%) increased due to Ni stress. However, CAT (21.59%) showed a decline (Figure 3 and Figure 4). AsA content declined by 21.91% and GSH increased by 12.05% due to Ni stress, whereas SNP at 100 µM increased AsA and GSH by 15.18 and 20.92% respectively (Figure 4C,D). Exogenous application of SNP (100 µM) ameliorated decline in AsA content by 17.49% over the Ni-stressed plants and caused further (11.66%) enhancement in GSH accumulation (Figure 4C,D).

### 2.6. Exogenous Application of NO Reduces Methylglyoxal by Upregulation Glyoxalase I Activity

**Figure 5:**
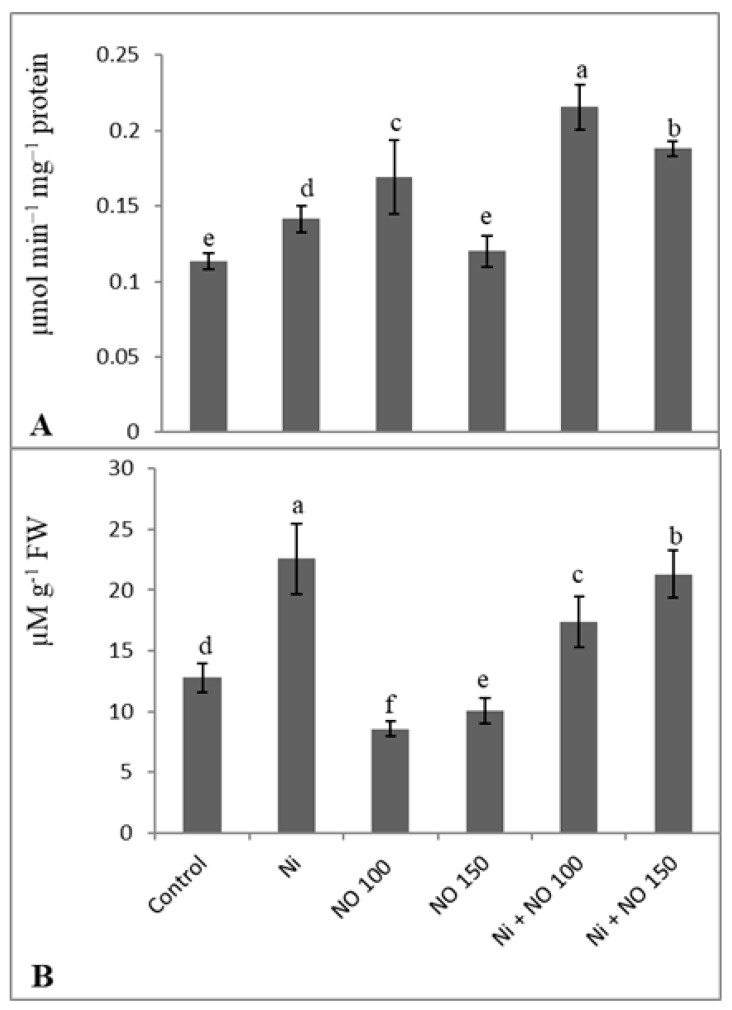
Effect of Ni (100 mg kg^−1^ soil) on (**A**) glyoxalase I activity and (**B**) methylglyoxal (MG) content in eggplant (*Solanum melongena* L.) treated with nitric oxide (100 and 150 µM SNP). Data presented is mean (±SE) of three replicates and bars with different letters denote significant difference at *p* ≤ 0.05. Control = 0 mg kg^−1^ soil.

Seedlings stressed with Ni showed increased accumulation of MG compared to the control and NO-treated ones. Relative to control MG was increased by 43.43% due to Ni stress while as was declined by 23.04% in Ni + 100 µM SNP-treated plants over the Ni-stressed. SNP (100 µM) supplemented plants exhibited significant increase (33.07%) in the activity of glyoxalase I. The percent increase in glyoxalase I activity was 19.81% due to Ni stress, which was further enhanced by the application of SNP causing significant decline in MG accumulation (Figure 5A,B).

### 2.7. Effect of Exogenous NO and NO Scavenger (PTIO) on Alleviation of Ni Stress

**Figure 6:**
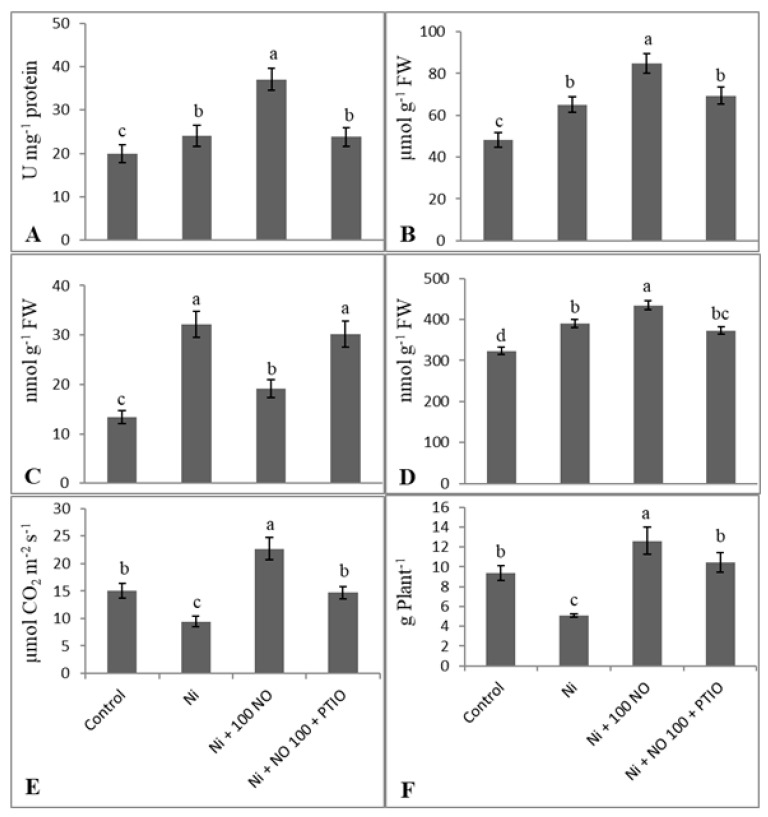
Effect of Ni (100 mg kg^−1^ soil), SNP (100 µM) and NO scavenger (2-phenyl-4,4,5,5-tetramethylimidazoline-1-oxyl-3-oxide (PTIO) on (**A**) superoxide dismutase activity, (**B**) proline, (**C**) hydrogen peroxide, (**D**) reduced glutathione, (**E**) photosynthesis, and (**F**) plant dry mass in eggplant (*Solanum melongena* L.). Data presented is mean (±SE) of three replicates and bars with different letters denote significant difference at *p* ≤ 0.05. Control = 0 mg kg^−1^ soil.

Application of NO scavenger, PTIO, along with SNP was investigated and considerable reversal of parameters studied was observed. PTIO treated seedlings exhibited almost complete elimination of NO mediated effects (Figure 6). Relative to Ni + SNP-treated plants, Ni + SNP + PTIO-treated plants exhibited decline of 21.92% in shoot length, 17.41% in plant dry weight, 35.24% in net photosynthesis, 14.27% in GSH, 55.68% in SOD activity, and 18.06% in proline accumulation. Such results of SNP + PTIO treated plants were comparable to control plants for shoot length, plant dry weight, and net photosynthesis; however, increase in GSH, H_2_O_2_, SOD, and proline accumulation was maintained almost equal to Ni-stressed plants. Contrary to Ni + SNP-treated and control plants, the accumulation of H_2_O_2_ increased by 36.81% and 55.67%, depicting the involvement of SNP application in mitigation of Ni induced oxidative damage (Figure 6).

## 3. Discussion

During the past few decades, metal and metalloid pollution has witnessed a considerable increase due to rapid industrial growth. Among the toxic metals, Ni has been identified as key constituents accumulating in considerable concentrations, thereby significantly declining the growth and yield of maximum crop plants. Higher concentrations of Ni impart toxic effects by inducing the excessive generation of ROS, resulting in a significant decline in growth and plant functioning [5]. Therefore, lessening the adverse effects of Ni toxicity by adopting efficient management techniques to strengthen the indigenously existing tolerance mechanisms becomes essential, and in this connection, we examined the role of exogenously supplied SNP in mitigating the Ni-mediated decline in growth. It was observed that exogenously SNP mitigated the Ni-mediated decline more obviously at lower concentrations (100 µM) affectively compared to 150 µM SNP. Availability of Ni restricts plant growth by declining the rate of cellular division and elongation [23]. Exogenously supplied SNP has been reported to help different crop species to withstand growth restrictions under stress, and such changes have been ascribed to its key role in signaling and elicitation of tolerance mechanisms [18,24]. Applied SNP mitigates the Ni-induced deleterious effects by interacting with other molecules, including phytohormones like salicylic acid [25]. In the present study, the beneficial role of applied NO was confirmed using the scavenger, PTIO (Figure 6) depicting its active involvement in cellular division, differentiation, and elongation. Increased growth and subsequent amelioration of Ni-mediated damaging effects in SNP (100 µM)-treated seedlings were seen correlated with significant enhancement in the uptake of N, K, and Ca, and decline in Ni accumulation. It has been reported that N, K, and Ca form key components of a cellular system and are involved in the regulation of various plant functions including enzyme activity, photosynthesis, stress signaling, and tolerance [26,27,28].

Applied NO increased chlorophyll and carotenoid synthesis, reflectied in enhanced photosynthetic rate and gas exchange, and, in addition, ameliorated the Ni-induced decline. Reduced growth and chlorophyll synthesis due to Ni stress have earlier been demonstrated [4,5]. Abiotic stresses trigger chlorophyll degradation by activating chlorophyllase activity and down-regulating chlorophyll biosynthesis [13,29]. In addition, Ni-mediated decline in Mg [25], and N [5] uptake may have contributed significantly to reduced synthesis of chlorophyll and Rubisco reflecting in declined photosynthesis and production of photoassimilates therefore restricting the growth. In the present study, it was observed that the positive effect of NO on photosynthesis was equally reversed due to PTIO treatment depicting its involvement in the alleviation of Ni-mediated photosynthetic inhibition. From the present study, it may be inferred that appropriate supplementation of SNP potentially alleviated the ill effects of Ni on photosynthesis by regulating chlorophyll and gas exchange attributes. Earlier SNP has been reported to regulate polyamine mediated stomatal functioning in Arabidopsis [30]. The concentration-dependent effect of SNP on the stomatal parameters appears in the present study and may have contributed to photosynthetic regulation under Ni stress condition. In addition to this, SNP-mediated modulations in non-stomatal parameters may have also significantly contributed to photosynthetic regulation [5]. In Ni-stressed *Brassica napus*, applied NO has been reported to significantly ameliorate the declined chlorophyll synthesis [31]. Plants deficient in NO production exhibit increased expression of chlorophyll degrading genes [32] and maintaining NO levels through exogenous application can potentially prevent such deleterious effects.

We observed a significant decline in accumulation of ROS like H_2_O_2_, lipoxygenase activity, and lipid peroxidation due to NO application. Stress-mediated increase in lipoxygenase activity intensifies the peroxidation of membranes and therefore hampering their functioning [9]. Greater H_2_O_2_ and lipid peroxidation due to Ni stress have been earlier reported in *Brassica napus* [31] and *Brassica juncea* [5]. However, exogenous NO at 100 µM significantly averted the oxidative damage to membranes by allowing reduced accumulation of H_2_O_2_. Stress-mediated increased the generation of H_2_O_2_ influences the structural and functional integrity of chloroplast and mitochondria, and NO application potentially lessens the damage by upregulating the antioxidant system [17,18]. In addition to H_2_O_2_ being a substrate for membrane peroxidases, this can result in stiffening of cell walls, thereby restricting cellular elongation [33]. The efficient scavenging system prevents diffusion of ROS into other cellular spaces leading to the prevention of further damage to cells and in the present study reduced oxidative damage in seedlings treated with exogenous SNP was almost entirely nullified by the NO scavenger (Figure 6) justifying the role of NO in the prevention of oxidative damage under Ni stress.

In the present study, SNP-mediated reduction in lipoxygenase activity reflects reduced lipolytic activity and oxidation of membrane-bound fatty acids, thereby restricting the lipid peroxidation. Reduced lipoxygenase activity and lipid peroxidation due to exogenously applied NO has been ascribed to the strengthening of ROS eliminating system [9], and in the present study, the involvement of SNP in upregulation of ROS neutralizing mechanisms, including antioxidant system and osmolyte accumulation, was confirmed using PTIO (Figure 6). Increased activity of SOD and accumulation of GSH and proline under Ni due to NO application was inhibited due to NO scavenger. Effective ROS scavenging system protects the cellular functioning from the deleterious effects of excess ROS [13,14,34]. SNP application strengthened the ROS elimination mechanisms by upregulating the antioxidant system functioning and the content of redox components, including AsA and GSH with apparent effects at 100 µM NO. The authors of [25,35] also reported increased activity of antioxidant enzymes due to NO application. Increased antioxidant functioning due to NO application prevents the oxidative effects on the structure and function of chloroplast thylakoids [17]. In the present study, NO-mediated enhancement in the APX and GR activities and synthesis of AsA and GSH reflects in improved electron transport through maintenance of NADP [12]. Such effects of exogenous SNP were evident as increased photosynthetic rate. Maintenance of redox constituents like AsA and GSH through upregulation of APX and GR activities results in the flow of electrons to molecular oxygen in the chloroplasts therefore preventing the generation of superoxide radical [36]. Ni accumulation downregulated CAT and declined AsA; however, exogenous SNP application resulted in significant enhancement providing further strength to tolerance mechanism to withstand Ni stress.

Accumulation of MG was significantly declined by NO application through upregulation of glyoxalase I, and similar effects were maintained under Ni stress. MG is intermediate of triose phosphates in glycolysis and also accumulates by the leakage of 1,2-enediolate intermediate from the active sites of triosephosphate isomerase [37]. Increased MG accumulation inhibits cellular proliferation and intensifies protein degradation by modifying cysteine and lysine residues, adducting within the guanyl nucleotides and weakens the antioxidant system [38]. In the MG detoxification pathway, glyoxalase-I forms the key component and carries frontline reaction utilizing GSH [9]. In the current study, exogenous SNP enhanced GSH synthesis, assisting glyoxalase I to maximally detoxify Ni stress generated MG. The functioning of glyoxalase depends on the GSH concentrations in the cells [39]. Increased accumulation of MG can lead to irreversible disarray, cell death, and mutations [40]. Compared to Ni-stressed and control, SNP-treated exhibited declined accumulation of MG, thereby preventing the possible toxic effects of MG on cellular stability. Earlier upregulation of glyoxalase-I due to kinetin [12] and NO [9] application has been demonstrated to mitigate salinity and cadmium mediated growth decline. However, reports discussing the effect of SNP on glyoxalase under Ni stress are not available.

Tolerance mechanisms against Ni toxicity were further strengthened by higher proline and glycine betaine accumulation in SNP supplemented seedlings attaining maximum accumulation with 100 M µM SNP. Organic osmolytes maintain tissue water content and activate down-stream stress signaling for improved tolerance [11,41]. Proline scavenges ROS and thereby lessens the oxidative effects [41], and NO-induced enhancement in their accumulation may have contributed to the redox homeostasis and thereby assuaging the Ni-induced growth restrictions by elevating the photosynthetic efficiency. Higher proline and glycine betaine have been reported to significantly ameliorate the stress-mediated photoinhibition [42,43], leading to maintenance the tissue and cellular osmotic balance by improving the water influx [11]. Earlier, the authors of [44] demonstrated improved carboxylase activity of Rubisco, due to enhanced proline accumulation and SNP-mediated increase in proline and glycine betaine, may have prevented Ni-mediated oxidative damage by efficient elimination of ROS, maintenance of redox balance, and osmolarity.

## 4. Materials and Methods 

### 4.1. Plant Growth and Stress Treatments

Experiments were conducted at the Department of Botany, Faculty of Science, Cairo University, Giza, Egypt during February and April 2018. Healthy eggplant (*Solanum melongena* L.) seeds were sterilized in 5% NaOCl for 10 min and were sown in a tray containing nutrient-rich substrate. After germination seedlings were grown for twenty days and were regularly irrigated. Twenty-day-old seedlings were transferred into pots (27 cm diameter) filled with soil and vermicompost (4:2). The soil used was having a pH of 7.2 and available concentration of N, P, and K as 59.11, 25.33, and 67.20 mg kg^−1^ soil, respectively. Pots were divided into two groups and were supplemented with 0 and 100 mg Ni kg^−1^ soil. NiSO_4_ was used as Ni salt and was thoroughly mixed with soil at the time of pot filling. Five days after successful seedling establishment, two healthy seedlings were maintained in every pot. A solution of 100 and 150 µM SNP (as sodium nitroprusside) was sprayed onto the foliage with a hand sprayer using teepol (0.1%) as surfactant twice a week for 25 days. Therefore, the detailed experimental treatments included: (i) control, (ii) Ni, (iii) 100 µM SNP, (iv) 150 µM SNP, (v) Ni + 100 µM SNP, and (iv) Ni + 150 µM SNP. To confirm the involvement of NO in Ni tolerance another experiment was laid, and plants were treated with NO scavenger (2-phenyl-4,4,5,5-tetramethylimidazoline-1-oxyl-3-oxide (PTIO, Sigma) along with 100 µM SNP to test the involvement of NO in amelioration of Ni triggered growth retardations. The chemistry of PTIO mediated NO scavenging can be seen elsewhere [45] and has been used widely in plant science research [35,46]. In both experiments, four replicates were taken for each treatment and arranged in completely randomized block design. Fifty days old seedlings (twenty days after Ni and SNP treatment or PTIO) were analyzed for certain physiological and biochemical parameters as discussed below

### 4.2. Estimation of Photosynthetic Pigments, Photosynthesis and Gas Exchange Parameters

For estimation of chlorophyll and carotenoids fresh 100 mg leaf tissue was extracted in 80% acetone using pestle and mortar. After centrifugation at 3000 g for 20 min, the supernatant was read at 480, 645, and 663 nm [47].

The gas exchange parameters including stomatal conductance (gs), intercellular CO_2_ concentration (Ci) and net photosynthetic rate (PN) were measured using infrared gas analyzer (CID-340, Photosynthesis System, Bio-Science, Washington, USA).

### 4.3. Estimation of Leaf Water Content, Proline, and Glycine Betaine

Relative water content (RWC) in leaves was estimated as follows [48]. Uniform sized leaf discs were punched from fresh leaf tissue and their fresh weight recorded (FW). Same leaf discs were kept in distilled water for 1 h to record turgid weight (TW) followed by drying at 70 °C for 24 h in the oven and dry weight (DW) was recorded. RWC was calculated by using the following formula.

(1) $$\mathrm{RWC}\%=\frac{\mathrm{FW}-\mathrm{DW}}{\mathrm{TW}-\mathrm{DW}}\times 100$$

Proline was extracted from 500 mg dry powdered leaf sample in 3% sulphosalicylic acid. The extract was centrifuged at 3000g for 20 min, and 2 mL supernatant was incubated at 100 °C for 1 h with glacial acetic acid and ninhydrin reagent in a water bath. Samples were cooled on ice, and proline was separated using toluene. Optical density was recorded at 520 nm [49].

Glycine betaine (GB) was estimated following the method of [50]. After extracting the dry powdered samples in distilled water, the filtered extract was diluted by 2N H_2_SO_4_. An appropriate aliquot was mixed with cold KI-I2 reagent and centrifuged at 10,000g for 15 min. The supernatant was aspirated, and periodide crystals were dissolved in 1,2-dichloromethane. After two hours, the absorbance was recorded at 365 nm, and GB content was calculated using the standard of glycine betaine.

### 4.4. Measurement of Membrane Stability Index, Hydrogen Peroxide, Lipid Peroxidation, and Lipoxygenase

For determination of membrane stability index (MSI), the method described by Sairam et al. (1997) was followed, and calculations were done using the following formula.

MSI = [1 − (C1/C2)] × 100(2)

Lipid peroxidation was determined by macerating 100 mg fresh leaf tissue in 1% trichloroacetic acid (TCA). After centrifugation at 10,000 g for 5 min, 1.0 mL supernatant heated at 95 °C with 0.5% thiobarbituric acid for half an hour. After cooling on ice bath samples were centrifuged at 5000g for 5 min, and absorbance was measured at 532 and 600 nm [51].

Hydrogen peroxide was determined by homogenizing 500 mg fresh tissue in 0.1% TCA, and the extract was centrifuged at 12,000g for 15 min; 0.5 mL of potassium phosphate buffer (pH 7.0) and 1 mL of potassium iodide were added to 0.5 mL of supernatant after thorough mixing, and absorbance was taken at 390 nm [52].

Lipoxygenase (LOX; EC, 1.13.11.12) activity was assayed by adopting the method in [53]. Linoleic acid was used as a substrate and change in optical density was monitored at 234 nm. The activity was expressed as units mg^−1^ protein, and extinction coefficient of 25 mM^−1^ cm^−1^ was used for calculation.

### 4.5. Assay of Glyoxalase I and Content of Methylglyoxal

For assaying glyoxylase I (EC: 4.4.1.5) activity 500 mg fresh tissue was macerated in cold potassium phosphate buffer (50 mM; pH 7.0) containing KCl (10 mM), β-mercaptoethanol (5 mM), ascorbate (1 mM) and glycerol(10%). Homogenate was centrifuged at 11,500g for 15 min, and the supernatant was used as enzymes source. Change in absorbance was recorded for 2 min at 240 nm in an assay mixture containing GSH (100 mM), 0.1 M phosphate buffer, 16 mM MgSO_4_, and distilled water. The reaction was initiated by addition of 35 mM methylglyoxal [54]. An extinction coefficient of 3.37 mM^−1^ cm^−1^ was used for calculation and expressed as μmol min^−1^ mg^−1^ protein.

The method described in [55] was followed or estimation of methylglyoxal leaf tissue was extracted in 5% perchloric acid, and the extract was centrifuged for 10 min at 11,000g. Charcoal was added to decolorise the supernatant and neutralized by potassium carbonate. The supernatant was mixed with sodium dihydrogen phosphate and N-acetyl-L-cysteine and left for 10 min. Content of N-α-acetyl-S-(1-hydroxy-2-oxo-prop-1-yl) cysteine formed was recorded at 288 nm.

### 4.6. Assay of Antioxidant Enzymes

Antioxidant enzymes were extracted by homogenizing 1 gm fresh leaf tissue in cold phosphate buffer (50 mM, pH 7.0) supplemented with 1% polyvinyl pyrolidine and 1 mM EDTA using chilled pestle and mortar. After centrifugation for 20 min at 15,000g at 4 °C, supernatant was collected and used as an enzyme source. Protein was determined in the supernatant using the method in [56].

Superoxide dismutase (SOD, EC 1.15.1.1) activity was assayed in accordance with the method of [57]. Inhibition of photochemical reduction of nitroblue tetrazolium chloride (NBT) at 560 nm was recorded in an assay mixture containing sodium phosphate buffer (50 mM, pH 7.5), 100 μL of EDTA, L-methionine (13 mM), NBT (75 μM), riboflavin (75 μM), and 100 μL enzyme extract. After illumination of 15 min reaction was terminated by switching off the light and absorbance was taken 560 nm against the non-illuminated blank. One unit of SOD was considered an amount of enzyme causing 50% inhibition in the photoreduction of NBT and activity was expressed as EU mg-1 protein.

Catalase (CAT, EC1.11.1.6) activity was assayed by monitoring change in optical density at 240 nm for 2 min. Reaction mixture contained phosphate buffer (100 mM; pH 7.0), 0.1 mM EDTA, H_2_O_2_, and 100 µL enzyme. The extinction coefficient of 39.4 mM^−1^cm^−1^ was used for calculation and activity expressed as U mg^−1^ protein [58].

GST (EC: 2.5.1.18) was assayed by recording the absorbance at 340 nm for 2 min in 1 mL assay mixture having 100 mM Tris-HCl buffer (pH 6.5), GSH (1.5 mM), 1-chloro-2, 4-dinitrobenzene (1 mM), and enzyme. For calculation extinction, a coefficient of 9.6 mM^−1^cm^−1^ was used.

The method in [59] was followed for determining the activity of ascorbate peroxidase (APX, EC 1.11.1.11), and absorbance was recorded at 290 nm for 3 min. One milliter of assay mixture contains phosphate buffer (50 mM; pH 7.0), 0.5 mM ascorbic acid, hydrogen peroxide, and enzyme extract (100 μL). For calculation, an extinction coefficient of 2.8 mM^−1^ cm^−1^ was used.

For glutathione reductase (GR; EC 1.6.4.2) activity method of [60] was adopted and change in optical density was observed at 340 nm for 2 min. The assay mixture was having phosphate buffer (50 mM, pH 7.8), nicotinamide adenine dinucleotide phosphate (NADPH, 0.12 mM), oxidized glutathione (GSSG, 0.5 mM) and 100 μL enzyme extract. The extinction coefficient of 6.2 mM^−1^ cm^−1^ was used for calculation and activity was expressed as l mol NADPH oxidized min−1 (units mg^−1^ protein).

### 4.7. Ascorbate and Reduced Glutathione Estimation

Ascorbate (AsA) content was measured by homogenizing 100 mg fresh plant material in TCA (6%). After centrifugation for 10 min at 5000 g, supernatant was mixed with 2% dinitrophenylhydrazine (DNPH, prepared in 9 M H_2_SO_4_) and 10% thiourea. After boiling in a water bath for 15 min samples were cooled and 5 mL of cold 80% H_2_SO_4_ was added. Optical density was taken at 530 nm, and calculation was done from a standard curve of ascorbate [61].

For determination of reduced glutathione (GSH) fresh 100 mg fresh leaf tissue was macerated in phosphate buffer (pH 8.0) followed by centrifugation for 15 min at 3000g. Five-hundred milliliters of supernatant and 500 µL of 5,5-dithiobis-2-nitrobenzoic acid were mixed and read at 412 nm after 10 min. Standard curve of GSH was used for calculation [62].

### 4.8. Estimation of Ions

Estimation of K and Ca was done flame photometrically after digesting 1 gm dried samples using H_2_SO_4_ and HClO_4_. Nitrogen was estimated following the method in [63].

Nickel (Ni^2+^) was estimated using atomic absorption Spectrophotometer (Shimadzu AA-6300, Japan) after dried samples were digested in HNO_3_ and HClO_4_ solution (3:1; v/v) [64].

### 4.9. Statistical Analysis

Data is the mean (±SE) of three replicates, and Duncan’s Multiple Range Test was performed using one-way ANOVA for determining the least significant difference (LSD) at *p* < 0.05 as explained in [65].

## 5. Conclusions

Ni stress restricted the growth of eggplant by triggering oxidative damage to membranes and inhibiting the photosynthesis. Exogenous NO (in the form of SNP, as the effects of SNP are attributed to NO) at 100 µM effectively averted the damaging effects of Ni by upregulating the antioxidant and glyoxalase system, resulting in reduced oxidative damage to membranes. Increased mineral uptake and synthesis of pigments due to exogenous NO application accompanied by efficient antioxidant functioning and osmolyte accumulation may have assisted eggplant seedlings to counteract the Ni toxicity. Therefore, from the present study, supplementation of optimal supplementation of NO for greater protection against Ni toxicity is advocated.

## Figures and Tables

**Table 1:** Effect of Ni (100 mg kg^−1^ soil) on shoot height, dry weight, leaf N, K, Ca, and Ni in eggplant (*Solanum melongena* L.) treated with nitric oxide (100 and 150 µM). Data presented is mean (±SE) of three replicates and bars with different letters denote significant difference at *p* ≤ 0.05.

	Control	Ni	100 µM SNP	150 µM SNP	Ni+100µM SNP	Ni+150µM SNP
Shoot height (cm)	43.8 ± 3.01c	31.0 ± 2.87e	52.0 ± 4.46a	48.2 ± 4.6ab	37.8 ± 3.2d	33.20 ± 3.40e
Shoot dry weight (g /plant)	7.3 ± 1.02c	4.9 ± 0.35e	12.6 ± 1.37a	10.4 ± 1.0b	9.1 ± 0.8b	6.73 ± 0.92d
Leaf Nitrogen (mg /g DW)	20.7 ± 2.05c	11.5 ± 1.69ef	27.7 ± 2.05a	23.3 ± 2.3b	16.3 ± 1.2d	13.53 ± 1.26e
Leaf potassium (mg /g DW)	22.5 ± 2.50b	12.5 ± 1.50e	29.7 ± 3.05a	28.2 ± 2.8a	17.1 ± 1.3c	15.03 ± 2.04d
Leaf calcium (mg /g DW)	5.8 ± 0.630b	3.0 ± 0.061d	7.1 ± 0.351a	6.2 ± 0.26ab	4.5 ± 0.3c	3.43 ± 0.294d
Leaf Nickel (mg /g DW)	0.0047 ± 0.0004d	3.09 ± 0.26a	0.0043 ± 0.0004d	0.0047 ± 0.0007d	2.15 ± 0.17c	2.84 ± 0.15b

**Table 2:** Effect of Ni (100 mg kg^−1^ soil) on total chlorophylls and carotenoids, net photosynthesis, intercellular CO_2_ concentration and stomatal conductance in eggplant (*Solanum melongena* L.) treated with nitric oxide (100 and 150 µM). Data presented is mean (±SE) of three replicates and bars with different letters denote significant difference at *p* ≤ 0.05.

	Control	Ni	100 µM SNP	150 µM SNP	Ni+100µM SNP	Ni+150µM SNP
Total chlorophyll (mg /g FW)	1.3 ± 0.088b	0.7 ± 0.025de	1.8 ± 0.065a	1.4 ± 0.07b	1.1 ± 0.04c	0.8 ± 0.018d
Carotenoids (mg /g FW)	0.3211 ± 0.01bc	0.2074 ± 0.01f	0.3945 ± 0.0064a	0.3348 ± 0.006b	0.3000 ± 0.002d	0.2279 ± 0.0042e
Net Photosynthesis (µmol CO_2_ m^−2^S^−1^)	16.3 ± 0.55c	8.8 ± 0.20f	25.0 ± 1.68a	17.9 ± 0.9b	13.3 ± 0.81d	10.3 ± 0.98e
Stomatal conductance (mmol m^−2^ S^−1^)	307.6 ± 12.42c	229.6 ± 8.50e	410.3 ± 11.1a	356.0 ± 10.5b	307.0 ± 9.5c	286.3 ± 8.62d
Intercellular CO_2_ concentration (µmol mol^−1^)	220.6 ± 7.37c	162.6 ± 5.85e	318.6 ± 7.02a	271.3 ± 6.5b	213.0 ± 6.2c	196.6 ± 7.09d

## References

[1] Shahzad B., Tanveer M., Rehman A., Cheema S.A., Fahad S., Rehman S., Sharma A. (2018). Nickel; whether toxic or essential for plants and environment—A review. Plant Physiol. Biochem..

[2] Chen C., Huang D., Liu J. (2009). Functions and toxicity of nickel in plants: Recent advances and future prospects. CLEAN–Soil Air Water.

[3] Mousavi H.Z., Seyedi S.R. (2011). Nettle ash as a low cost adsorbent for the removal of nickel and cadmium from wastewater. Int. J. Environ. Sci. Technol..

[4] Seregin I.V., Kozhevnikova A.D. (2006). Physiological role of nickel and its toxic effects on higher plants. Russ. J. Plant Physiol..

[5] Khan M.I.R., Khan N.A. (2014). Ethylene reverses photosynthetic inhibition by nickel and zinc in mustard through changes in PS II activity, photosynthetic nitrogen use efficiency, and antioxidant metabolism. Protoplasma.

[6] Sytar O., Kumar A., Latowski D., Kuczynska P., Strzalka K., Prasad M.N.V. (2013). Heavy metal-induced oxidative damage, defense reactions, and detoxification mechanisms in plants. Acta Physiol. Plant..

[7] Ventrella A., Catucci L., Piletska E., Piletsky S., Agostiano A. (2009). Interactions between heavy metals and photosynthetic materials studied by optical techniques. Bioelectrochemistry.

[8] Per T.S., Khan N.A., Masood A., Fatma M. (2016). Methyl Jasmonate Alleviates Cadmium-Induced Photosynthetic Damages through Increased S-Assimilation and Glutathione Production in Mustard. Front. Plant Sci..

[9] Nahar K., Hasanuzzaman M., Alam M.M., Rahman A., Suzuki T., Fujita M. (2016). Polyamine and nitric oxide crosstalk: Antagonistic effects on cadmium toxicity in mung bean plants through upregulating the metal detoxification, antioxidant defense and methylglyoxal detoxification systems. Ecotoxicol. Environ. Saf..

[10] El-Esawi M.A., Elkelish A., Elansary H.O., Ali H.M., Elshikh M., Witczak J., Ahmad M. (2017). Genetic transformation and hairy root induction enhance the antioxidant potential of *Lactuca serriola* L.. Oxid. Med. Cell. Longev..

[11] Ahanger M.A., Tyagi S.R., Wani M.R., Ahmad P. (2014). Drought tolerance: Role of organic osmolytes, growth regulators, and mineral nutrients. Physiological Mechanisms and Adaptation Strategies in Plants under Changing Environment.

[12] Ahanger M.A., Alyemeni M.N., Wijaya L., Alamri S.A., Alam P., Ashraf M., Ahmad P. (2018). Potential of exogenously sourced kinetin in protecting Solanum lycopersicum from NaCl-induced oxidative stress through up-regulation of the antioxidant system, ascorbate-glutathione cycle and glyoxalase system. PLoS ONE.

[13] Soliman M.H., Alayafi A.A.M., El Kelish A.A., Abu-Elsaoud A.M. (2018). Acetylsalicylic acid enhance tolerance of Phaseolus vulgaris L. to chilling stress, improving photosynthesis, antioxidants and expression of cold stress responsive genes. Bot. Stud..

[14] Elkeilsh A., Awad Y.M., Soliman M.H., Abu-Elsaoud A., Abdelhamid M.T., El-Metwally I.M. (2019). Exogenous application of β-sitosterol mediated growth and yield improvement in water-stressed wheat (*Triticum aestivum*) involves up-regulated antioxidant system. J. Plant Res..

[15] Arasimowicz M., Floryszak-Wieczorek J. (2007). Nitric oxide as a bioactive signalling molecule in plant stress responses. Plant Sci..

[16] Asgher M., Per T.S., Masood A., Fatma M., Freschi L., Corpas F.J., Khan N.A. (2017). Nitric oxide signaling and its crosstalk with other plant growth regulators in plant responses to abiotic stress. Environ. Sci. Pollut. Res..

[17] Fatma M., Masood A., Per T.S., Khan N.A. (2016). Nitric oxide alleviates salt stress inhibited photosynthetic performance by interacting with sulfur assimilation in mustard. Front. Plant Sci..

[18] Ahmad P., Ahanger M.A., Alyemeni M.N., Wijaya L., Alam P. (2018). Exogenous application of nitric oxide modulates osmolyte metabolism, antioxidants, enzymes of ascorbate-glutathione cycle and promotes growth under cadmium stress in tomato. Protoplasma.

[19] Yeung A.W.K., Tzvetkov N.T., El-Tawil O.S., Bungǎu S.G., Abdel-Daim M.M., Atanasov A.G. Antioxidants: Scientific Literature Landscape Analysis. https://www.hindawi.com/journals/omcl/2019/8278454/.

[20] Khan M.N., Siddiqui M.H., Mohammad F., Naeem M. (2012). Interactive role of nitric oxide and calcium chloride in enhancing tolerance to salt stress. Nitric Oxide.

[21] Abdel-Daim M.M., Zakhary N.I., Aleya L., Bungǎu S.G., Bohara R.A., Siddiqi N.J. (2018). Aging, Metabolic, and Degenerative Disorders: Biomedical Value of Antioxidants. Oxidative Med. Cell. Longev..

[22] Mahmoud E.K., Ghoneim A.M. (2016). Effect of polluted water on soil and plant contamination by heavy metals in El-Mahla El-Kobra, Egypt. Solid Earth.

[23] Demchenko N.P., Kalimova I.B., Demchenko K.N. (2005). Effect of nickel on growth, proliferation, and differentiation of root cells in Triticum aestivum seedlings. Russ. J. Plant Physiol..

[24] Ahanger M.A., Akram N.A., Ashraf M., Alyemeni M.N., Wijaya L., Ahmad P. (2017). Signal transduction and biotechnology in response to environmental stresses. Biol. Plant..

[25] Kotapati K.V., Palaka B.K., Ampasala D.R. (2017). Alleviation of nickel toxicity in finger millet (Eleusinecoracana L.) germinating seedlings by exogenous application of salicylic acid and nitric oxide. Crop J..

[26] Ahanger M.A., Agarwal R.M. (2017). Salinity stress induced alterations in antioxidant metabolism and nitrogen assimilation in wheat (Triticum aestivum L) as influenced by potassium supplementation. Plant Physiol. Biochem..

[27] Iqbal N., Umar S., Khan N.A. (2015). Nitrogen availability regulates proline and ethylene production and alleviates salinity stress in mustard (*Brassica juncea*). J. Plant Physiol..

[28] Elkelish A.A., Alnusaire T.S., Soliman M.H., Gowayed S., Senousy H.H., Fahad S. (2019). Calcium availability regulates antioxidant system, physio-biochemical activities and alleviates salinity stress mediated oxidative damage in soybean seedlings. J. Appl. Bot. Food Qual..

[29] Dalal V.K., Tripathy B.C. (2012). Modulation of chlorophyll biosynthesis by water stress in rice seedlings during chloroplast biogenesis. Plant Cell Environ..

[30] Agurla S., Gayatri G., Raghavendra A.S. (2018). Polyamines increase nitric oxide and reactive oxygen species in guard cells of Arabidopsis thaliana during stomatal closure. Protoplasma.

[31] Kazemi N., Khavari-Nejad R.A., Fahimi H., Saadatmand S., Nejad-Sattari T. (2010). Effects of exogenous salicylic acid and nitric oxide on lipid peroxidation and antioxidant enzyme activities in leaves of Brassica napus L. under nickel stress. Sci. Hortic..

[32] Liu F., Guo F.-Q. (2013). Nitric Oxide Deficiency Accelerates Chlorophyll Breakdown and Stability Loss of Thylakoid Membranes during Dark-Induced Leaf Senescence in Arabidopsis. PLoS ONE.

[33] Díaz J., Bernal A., Pomar F., Merino F. (2001). Induction of shikimate dehydrogenase and peroxidase in pepper (*Capsicum annuum* L.) seedlings in response to copper stress and its relation to lignification. Plant Sci..

[34] El-Esawi M.A., Al-Ghamdi A.A., Ali H.M., Ahmad M. (2019). Overexpression of *AtWRKY30* Transcription Factor Enhances Heat and Drought Stress Tolerance in Wheat (*Triticum aestivum* L.). Genes.

[35] Rizwan M., Mostofa M.G., Ahmad M.Z., Imtiaz M., Mehmood S., Adeel M., Dai Z., Li Z., Aziz O., Zhang Y. (2018). Nitric oxide induces rice tolerance to excessive nickel by regulating nickel uptake, reactive oxygen species detoxification and defense-related gene expression. Chemosphere.

[36] Noctor G., Foyer C.H. (1998). Ascorbate and glutathione: Keeping active oxygen under control. Annu. Rev. Plant Biol..

[37] Li Z.-G. (2016). Methylglyoxal and glyoxalase system in plants: Old players, new concepts. Bot. Rev..

[38] Martins A.M.T., Cordeiro C.A.A., Ponces Freire A.M.J. (2001). In situ analysis of methylglyoxal metabolism in Saccharomyces cerevisiae. FEBS Lett..

[39] Yadav S.K., Singla-Pareek S.L., Ray M., Reddy M.K., Sopory S.K. (2005). Methylglyoxal levels in plants under salinity stress are dependent on glyoxalase I and glutathione. Biochem. Biophys. Res. Commun..

[40] Yadav S.K., Singla-Pareek S.L., Sopory S.K. (2008). An overview on the role of methylglyoxal and glyoxalases in plants. Drug Metab. Drug Interact..

[41] Hayat S., Hayat Q., Alyemeni M.N., Wani A.S., Pichtel J., Ahmad A. (2012). Role of proline under changing environments. Plant Signal. Behav..

[42] Khan M.I.R., Asgher M., Khan N.A. (2014). Alleviation of salt-induced photosynthesis and growth inhibition by salicylic acid involves glycinebetaine and ethylene in mungbean (*Vigna radiata* L.). Plant Physiol. Biochem..

[43] Khan M.I.R., Nazir F., Asgher M., Per T.S., Khan N.A. (2015). Selenium and sulfur influence ethylene formation and alleviate cadmium-induced oxidative stress by improving proline and glutathione production in wheat. J. Plant Physiol..

[44] Sivakumar P., Sharmila P., PardhaSaradhi P. (2000). Proline Alleviates Salt-Stress-Induced Enhancement in Ribulose-1,5-Bisphosphate Oxygenase Activity. Biochem. Biophys. Res. Commun..

[45] Goldstein S., Russo A., Samuni A. (2003). Reactions of PTIO and Carboxy-PTIO with·NO,·NO2, and. J. Biol. Chem..

[46] He H., Oo T.L., Huang W., He L.-F., Gu M. (2019). Nitric oxide acts as an antioxidant and inhibits programmed cell death induced by aluminum in the root tips of peanut (*Arachis hypogaea* L.). Sci. Rep..

[47] Arnon D.I. (1949). Copper enzymes in isolated chloroplasts. Polyphenoloxidase in Beta vulgaris. Plant Physiol..

[48] Smart R.E., Bingham G.E. (1974). Rapid estimates of relative water content. Plant Physiol..

[49] Bates L.S., Waldren R.P., Teare I.D. (1973). Rapid determination of free proline for water-stress studies. Plant Soil.

[50] Grieve C.M., Grattan S.R. (1983). Rapid assay for determination of water soluble quaternary ammonium compounds. Plant Soil.

[51] Heath R.L., Packer L. (1968). Photoperoxidation in isolated chloroplasts: I. Kinetics and stoichiometry of fatty acid peroxidation. Arch. Biochem. Biophys..

[52] Velikova V., Yordanov I., Edreva A. (2000). Oxidative stress and some antioxidant systems in acid rain-treated bean plants. Plant Sci..

[53] Doderer A., Kokkelink I., van der Veen S., Valk B.E., Schram A., Douma A.C. (1992). Purification and characterization of two lipoxygenase isoenzymes from germinating barley. Biochim. Biophys. Acta (BBA)-Protein Struct. Mol. Enzymol..

[54] Hasanuzzaman M., Hossain M.A., Fujita M. (2011). Nitric oxide modulates antioxidant defense and the methylglyoxal detoxification system and reduces salinity-induced damage of wheat seedlings. Plant Biotechnol. Rep..

[55] Wild R., Ooi L., Srikanth V., Münch G. (2012). A quick, convenient and economical method for the reliable determination of methylglyoxal in millimolar concentrations: The N-acetyl-l-cysteine assay. Anal. Bioanal. Chem..

[56] Lowry O.H., Rosebrough N.J., Farr A.L., Randall R.J. (1951). Protein measurement with the Folin phenol reagent. J. Biol. Chem..

[57] Beyer W.F., Fridovich I. (1987). Assaying for superoxide dismutase activity: Some large consequences of minor changes in conditions. Anal. Biochem..

[58] Luck H., Bergmeyer J., Grabi M. (1974). Catalase in Methods of Enzymatic Analysis.

[59] Nakano Y., Asada K. (1981). Hydrogen peroxide is scavenged by ascorbate-specific peroxidase in spinach chloroplasts. Plant Cell Physiol..

[60] Foyer C.H., Fletcher J.M. (2001). Plant antioxidants: Colour me healthy. Biologist.

[61] Mukherjee S.P., Choudhuri M.A. (1983). Implications of water stress-induced changes in the levels of endogenous ascorbic acid and hydrogen peroxide in Vigna seedlings. Physiol. Plant..

[62] Ellman G.L. (1959). Tissue sulfhydryl groups. Arch. Biochem. Biophys..

[63] Subbaiah B.V. (1956). A rapid procedure for estimation of available nitrogen in soil. Curr. Sci..

[64] Sagner S., Kneer R., Wanner G., Cosson J.-P., Deus-Neumann B., Zenk M.H. (1998). Hyperaccumulation, complexation and distribution of nickel in Sebertiaacuminata. Phytochemistry.

[65] Elansary H.O., Szopa A., Kubica P., Ekiert H., Ali H.M., Elshikh M.S., Abdel-Salam E.M., El-Esawi M., El-Ansary D.O. (2018). Bioactivities of traditional medicinal plants in Alexandria. Evid. Based. Complement. Alternat. Med..

